# The Effect of Voluntary Exercise on Gut Microbiota in Partially Hydrolyzed Guar Gum Intake Mice under High-Fat Diet Feeding

**DOI:** 10.3390/nu12092508

**Published:** 2020-08-19

**Authors:** Takafumi Aoki, Eri Oyanagi, Chihiro Watanabe, Nanako Kobiki, Suzuka Miura, Yuka Yokogawa, Hiromi Kitamura, Fusako Teramoto, Michael J. Kremenik, Hiromi Yano

**Affiliations:** 1 Graduate School of Health Science and Technology, Kawasaki University of Medical Welfare, Kurashiki, Okayama 701-0193, Japan; aoki.takafumi.54331@mw.kawasaki-m.ac.jp (T.A.); wd219005@kwmw.jp (C.W.); wd219003@kwmw.jp (S.M.); wd219004@kwmw.jp (Y.Y.); teramoto@mw.kawasaki-m.ac.jp (F.T.); 2 Department of Clinical Nutrition, Kawasaki University of Medical Welfare, Kurashiki, Okayama 701-0193, Japan; 3 Department of Health and Sports Science, Kawasaki University of Medical Welfare, Kurashiki, Okayama 701-0193, Japan; eri-oyanagi@mw.kawasaki-m.ac.jp (E.O.); aoisonophoto@gmail.com (N.K.); kremelin@mw.kawasaki-m.ac.jp (M.J.K.); 4 Department of Human Health, University of Marketing and Distribution Sciences, Kobe, Hyogo 651-2103, Japan; hiromi_kitamura@red.umds.ac.jp

**Keywords:** dietary fiber, wheel running, F/B ratio

## Abstract

Although dietary fiber treatment alters the gut microbiota and its metabolite production, it is unclear whether or not exercise habits can have a supplemental effect on changes in gut microbiota in dietary fiber-treated mice. To clarify the supplemental effect of voluntary exercise on gut microbiota in partially hydrolyzed guar gum (PHGG), which is a soluble dietary fiber, treated mice under high-fat diet (HFD) feeding, 4-week-old male C57BL/6J mice (*n* = 80) were randomly divided into two dietary groups: the control-diet (CD) and HFD. Then, each dietary group was treated with or without PHGG, and with or without wheel running. After the experimental period, measurement of maximal oxygen consumption, a glucose tolerance test and fecal materials collection for analysis of gut microbiota were carried out. Voluntary exercise load in PHGG treatment under HFD feeding showed the supplemental effect of exercise on obesity (*p* < 0.01) and glucose tolerance (*p* < 0.01). Additionally, in both CD and HFD groups, voluntary exercise accelerated the decrease in the Firmicutes/Bacteroidetes ratio in mice fed with PHGG (*p* < 0.01). These findings suggest that voluntary exercise might activate the prevention of obesity and insulin resistance more via change in gut microbiota in mice administrated with PHGG.

## 1. Introduction

The ingestion of dietary fiber is a potential prebiotic, and may lead to numerous health benefits on the host [1,2]. The main object of prebiotics is to stimulate the growth and activity of beneficial bacteria in the gastrointestinal tract (GT) [1,2]. Guar gum is one of these dietary fibers, and is classified in soluble and fermentable fibers. In an attempt to make guar gum more palatable, hydrolyzed guar gum (partially hydrolyzed guar gum (PHGG)) was produced resulting in a low viscosity/nonviscous. Thus, PHGG is classified as low viscous in fibers, but it is well fermented [1]. Unfortunately, the beneficial effect, such as improved glycemic control, is abolished when the guar gum is hydrolyzed to a nonviscous form [3]. In fact, dietary PHGG did not significantly affect plasma glucose levels in a rodent model of type 2 diabetes and patients with type 2 diabetes and metabolic syndrome [4,5]. However, PHGG could elicit constipation relief and modulate gut microbiota, which shows the potential to act as a dietary fiber for constipation treatment [6]. In addition, PHGG improves symptoms associated with irritable bowel syndrome [7], demonstrated to be beneficial in the treatment of cholera [8], small intestinal bacterial overgrowth [9], and pediatric functional gastrointestinal disorders [10]. PHGG treatment has been shown to reduce colonic mucosal damage in an animal colitis model [11]. Although this fermentable fiber is useful as a prebiotic, it was reported that in high-fat diet (HFD)-induced obese mice, guar gum intake did not attenuate the body mass gain [12], and also the suppressive effect of PHGG on body mass gain is slight [13].

On the other hand, it is well known that exercise contributes to attenuating hyperglycemia [14] and has other numerous health benefits [15]. In addition, recent reviews suggest that exercise, which alters gut microbiota composition and function, is supported by rodent studies, although the responses to exercise vary with the novelty, frequency, intensity, and duration of activity [16,17].

Each PHGG intake and exercise alters the gut microbiota and its metabolite production, such as short-chain fatty acids (SCFAs) [1,18]. Accordingly, it can be expected as a remedy for deterioration of the gut microbiota which is decreased diversity of the gut microbiota and increased harmful bacteria (dysbiosis) [19]. The 16S rRNA gene sequence data of gut microbiota in mice indicate that HFD induces not only obesity, but also the increased Firmicutes and Bacteroidetes ratio (F/B ratio) [20,21,22]. Although it was reported that the abundance of Firmicutes and Bacteroidetes in HFD-feeding mice did not change with or without guar gum intake [12], the alteration of F/B ratio after PHGG intake has not been investigated. In addition, it is unclear whether or not exercise has a supplemental effect on alterations in the gut microbiota with PHGG intake.

Therefore, the purpose of this study was to clarify the supplemental effect of voluntary exercise on gut microbiota in PHGG intake under the HFD feeding used as an animal model.

## 2. Materials and Methods

### 2.1. Animal and Experimental Design

Four-week-old male C57BL/6J mice (*n* = 80, CLEA Japan, Tokyo, Japan) were housed individually in cages under a controlled environment (22 ± 1 °C, 12:12-h light-dark cycle) and were randomly divided into 2 dietary groups: the Control Diet (CD) and HFD. Then, each dietary group was treated with (GCDC, *n* = 12 and GHFDC, *n* = 12) or without (CDC, *n* = 8 and HFDC, *n* = 8) PHGG (G) intake, with wheel running (W) (CDW, *n* = 8 and HFDW, *n* = 8), and with a combination of G and W (GCDW, *n*= 12 and GHFDW, *n* = 12). The mice were given ad libitum access to food and drinking water (Supplement Figure S1). The experiment involving the mice including the procedures performed, was approved by the Institutional Animal Care and Use Committee of Kawasaki University of Medical Welfare (No.18-010).

### 2.2. Diet and PHGG

The mice were fed a normal CD (D12450Jpx1, Research Diets, New Brunswick, NJ, USA) containing 10% fat, 20% protein, and 70% carbohydrates (of total calories), and HFD (D12492Gpx10, Research Diets, New Brunswick, NJ, USA) containing 60% fat, 20% protein, and 20% carbohydrates for 10 weeks. Both diets were cellulose free. Each diet also contained two conditions, are with 5.0% PHGG (Sun fiber^®^, Taiyo Kagaku Co., Ltd., Yokkaichi, Japan) and are without 5.0% PHGG.

### 2.3. Voluntary Exercise

As for voluntary wheel running, each mouse (in CDW, GCDW, HFDW and GHFDW groups) ran on a wheel (10 × 23 × 10 cm cage with wide 5.5 cm × 22 cm wheel, Natsume, Nagano, Japan) in its cage freely for 10 weeks.

### 2.4. Measurement of Maximum Oxygen Consumption ( $\overset{\cdot }{V}$O_2_max)

To clarify the effect of voluntary exercise on aerobic capacity, a part of each mouse (*n* = 8 in each group) was measured for $\overset{\cdot }{V}$O_2_max at 9–10 weeks during the experimental period by using a treadmill in a metabolic chamber [23]. All mice were accustomed to treadmill running for 5 days prior to the measurement. Each mouse spent 5 min running at 5 m/min followed by 5 min running at 10 m/min, and then velocity was increased by 1 m/min every 30 s at inclinations of 20°. $\overset{\cdot }{V}$O_2_max was defined as the point when the oxygen consumption showed leveling off despite constant increases in velocity, or when the mice maintained continuous contact with the shock grid for 5 s or were unable to or refused to run further.

### 2.5. Glucose Tolerance Test (GTT)

To clarify the effect of voluntary exercise on glycemic control in PHGG-treated mice under HFD feeding, after the mice fasted for 5 h, blood samples were collected from the tail vein, and then blood glucose levels were measured using the glucose monitoring device Accu-Chek, (Roche, Basel, Switzerland) immediately prior to glucose administration, and at 15, 30, 60 and 120 min after glucose administration (2 g/kg, i.p.). Each mouse was lightly anesthetized with the inhalant Isoflurane prior to the glucose administration [24].

### 2.6. Measurement Body Mass, Food Intake, Tissue Mass, and Secum Contents

Body mass and food intake were recorded every week. Two days after the 10 week experimental period, the mice were sacrificed under isoflurane anesthesia. The heart, soleus, and cecum were collected. Epididymal, subcutaneous, and visceral fats were collected and weighed individually, and the results showed the total amount as total body fat.

### 2.7. Fecal pH Measurement and Analysis of Gut Microbiota

At the 6th week, fecal materials were collected from mice that were as fresh as possible. The materials were diluted 2-3-fold (*w*/*v*) in distilled water and were homogenized by the homogenizer pestle. After calibrating the pH meter (twin pH B-212, Horiba, Ltd., Kyoto, Japan), the pH of these diluted samples was measured [25]. At the 10th week, feces from mice were collected for analysis of fecal microbiota. After changing to a sterilized cage, feces from mice were pooled by the cage, and feces were collected and were immediately snap-frozen in liquid nitrogen. Each fecal sample was carried out using 16S rRNA metagenomics analysis by next-generation sequencing (NGS) methods. 16S rRNA sequences from bacteria were analyzed by the Ribosomal Database Project (RDP Release 11 Update 4 May 26, 2015) [26], which was used for alignment and classification (97% similarity) of operational taxonomic units (OTUs). The OTUs counts were normalized by subsampling to the lowest number of OTUs found in the sample. The α- and β-diversities of the gut microbiota were analyzed using Quantitative Insight into Microbial Ecology (QIIME ver. 1.8.0) [27].

### 2.8. Analysis of Short-Chain Fatty Acids (SCFAs)

Cecal contents were collected at the sacrifice and immediately frozen. SCFAs concentrations were quantified by HPLC using a post column reaction with a Prominence CDD-10Avp conductivity detector (Shimadzu, Kyoto, Japan), tandemly arranged two columns (Shim-pack SCR-102(H); 300 mm × 8.0 mm ID), and a guard column a Shim-pack SCR-102(H) guard column (50 mm × 6.0 mm ID) as mentioned previously [28].

### 2.9. Statistical Analysis

The statistical analyses were performed using the IBM SPSS Statistics 23.0 for Windows software program. First, the effect of diet was analyzed between CDC vs. HFDC by nonparametric analysis using the Mann-Whitney U test. Second, in each dietary condition (control diet fed groups and high-fat diet fed groups, respectively), the data were analyzed using the Kruskal-Wallis test, and then a post hoc test was performed using the Mann-Whitney U test. *p*-values of <0.05 were considered to indicate statistical significance.

## 3. Results

**Figure 1:**
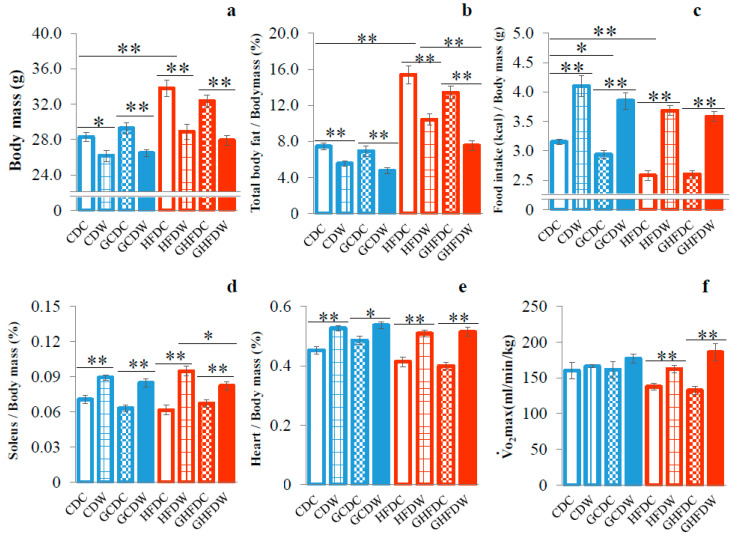
The supplemental effect of wheel running on body mass (**a**), total body fat (**b**), food intake (**c**), soleus muscle mass (**d**), heart mass (**e**), and $\overset{\cdot }{V}$O_2_max (**f**) in PHGG intake mice under CD and HFD feeding. The data in panels (**a**–**e**) were showed as percentage of body mass at the end of the experiment. Food intake showed as energy intake (kcal) per body mass. $\overset{\cdot }{V}$O_2_max was measured by measuring respiratory gas while running on a treadmill using a mass spectrometer (ALCO). CDC: control diet (CD) and sedentary control (C) (*n* = 8), GCDC: PHGG intake (G) and CDC (*n* = 12), CDW: CD and wheel running (W) (*n* = 8), GCDW (*n* = 12), HFDC: high-fat diet (HFD) and C (*n* = 8), GHFDC (*n* = 12), HFDW (*n* = 8), and GHFDW (*n* = 12). The values were expressed as the mean ± S.E.M. * *p* < 0.05 and ** *p* < 0.01.

Body mass in HFDC mice was significantly higher than that in CDC mice (*p* < 0.01, Figure 1a and Supplement Figure S2a). Accordingly, in this study, there was, at least, HFD-induced obesity in mice. In both CD- and HFD-fed conditions, wheel running attenuated body mass gain. Although the results for total body fat were also very similar to those for body mass, interestingly the body fat in GHFDW mice was significantly lower than that in HFDW (*p* < 0.01, Figure 1b). Food intake in HFDC was significantly lower than that in CDC (*p* < 0.01, Figure 1c). The food intake of the exercise groups (CDW, GCDW, HFDW and GHFDW) had remained high throughout the experiment under all conditions. Moreover, food intake in HFD mice had been similar to that in CD mice but HFD mice became obese (Supplement Figure S2b). In CD mice, PHGG decreased food intake (*p* < 0.05), but not in HFD mice. Moreover, wheel running accelerated food intake in both types of dietary mice treated with or without PHGG treatment (*p* < 0.01). In both dietary conditions, soleus muscle (Figure 1d) and heart (Figure 1e) mass in each wheel running group were significantly higher than that in each sedentary group (*p* < 0.05 or *p* < 0.01), although PHGG intake did not affect them. A high level of the $\overset{\cdot }{V}$O_2_max in the HFD fed condition was induced by wheel running (*p* < 0.01), but not by PHGG treatment (Figure 1f).

**Figure 2:**
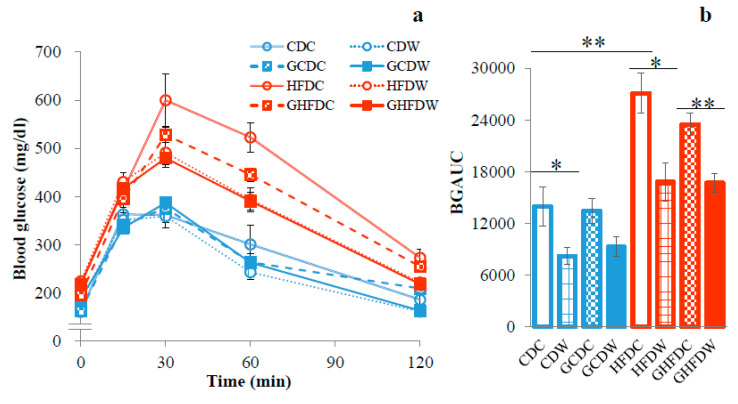
The supplemental effect of wheel running on blood glucose concentration and the incremental area under the curve (AUC) of blood glucose during GTT in PHGG intake mice under CD and HFD feeding. Time-dependent blood glucose level after glucose load (2 g/kg body mass) into the abdominal cavity (**a**) and the corresponding incremental blood glucose area under the curves (BGAUC) (**b**). The values were expressed as the mean ± S.E.M. * *p* < 0.05 and ** *p* < 0.01.

Figure 2a shows the changes in blood glucose concentration in GTT. BGAUC in HFDC was significantly higher than that in CDC mice (*p* < 0.01, Figure 2b). There was no difference between the BGAUC in HFDC and GHFDC mice. The BGAUC, however, was significantly attenuated by wheel running but had no effect on PHGG treatment (*p* < 0.05 and *p* < 0.01, respectively).

**Figure 3:**
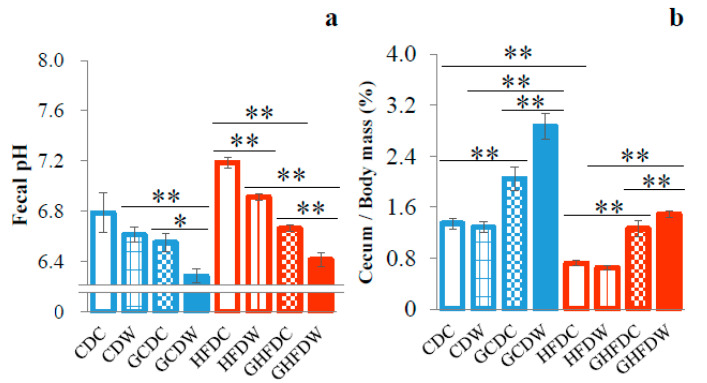
The supplemental effect of wheel running on fecal pH and cecum mass in PHGG intake mice under CD and HFD feeding. Fecal pH at 6 weeks (**a**) and cecum mass (**b**) showed as percentage of body mass after 10 weeks. The values were expressed as the mean ± S.E.M. * *p* < 0.05 and ** *p* < 0.01.

To explore the effect of the level of wheel running on gut microbiota in PHGG fed mice, we first analyzed the fecal pH in mice during the experimental period. Although the fecal pH showed no difference between in the CDC and HFDC mice, in both dietary groups of mice, combined between PHGG and wheel running was lower than that in each treated groups (GCDW vs. CDW and GCDC: *p* < 0.01 and *p* < 0.05, GHFDW vs. HFDW and GHFDC: *p* < 0.01, respectively, Figure 3a).

Cecum contents in HFDC were lower than that in CDC (*p* < 0.01). In both diet conditions (CD and HFD), the cecum content was significantly increased by PHGG intake (*p* < 0.01). Moreover, in the PHGG intake mice, the cecum content was significantly increased by wheel running (GCDC vs. GCDW: *p* < 0.01, and GHFDC vs. GHFDW: *p* < 0.01, Figure 3b).

**Figure 4:**
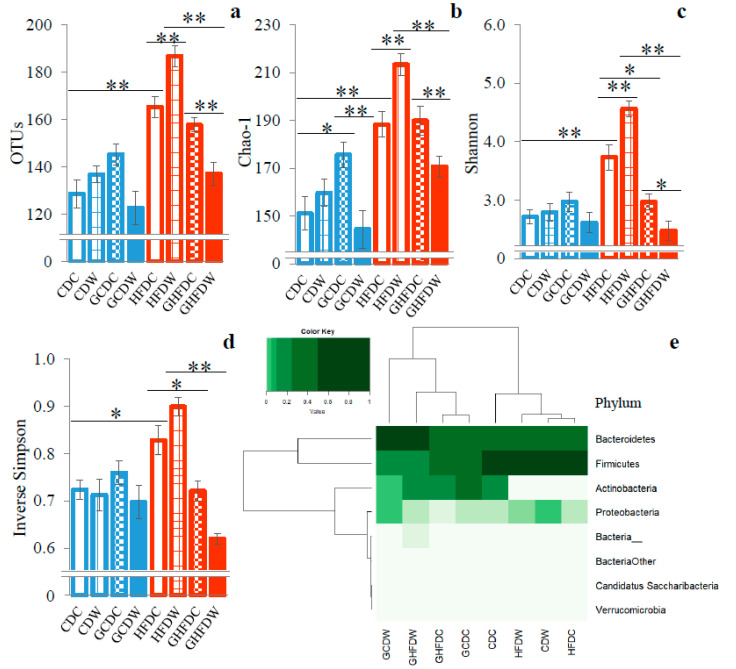
The supplemental effect of wheel running on bacterial diversities in the gut microbiota in PHGG intake mice under CD and HFD feeding. The OTUs (**a**), Chao-1 (**b**), Shannon (**c**), and inverse Simpson (**d**) indexes are shown as the α-diversities. The heatmap showed gut microbiota distribution at phyla level of taxon, and the dendrogram showed the clustering of phyla as the β-diversity (**e**). The values of α-diversities were expressed as the mean ± S.E.M. * *p* < 0.05 and ** *p* < 0.01.

The analyses of α- and β-diversities were performed to estimate bacterial richness and diversity (Figure 4). The scores of OTUs, Chao-1 and Shannon indexes other than Simpson index in the GHFDW mice were significantly lower than that in the GHFDC groups (*p* < 0.01, *p* < 0.01 and *p* < 0.05, Figure 4a–d). Furthermore, microbiota distribution (β-diversity) at phyla level of taxon contributing to 97% of sample variations was shown as the heatmap (Figure 4e). The dendrogram shows the clustering of phyla based on Ward’s hierarchical clustering method. Remarkably, all PHGG-intake groups clustered separately from the non-PHGG-intake groups.

**Figure 5:**
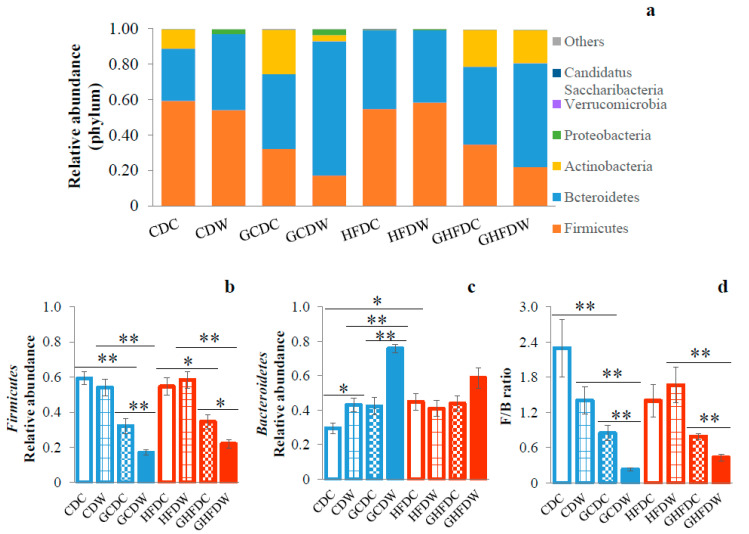
The supplemental effect of wheel running on gut microbiota at phylum level in PHGG intake mice under CD and HFD feeding. Relative abundance at phylum level (**a**), Firmicutes (**b**), Bacteroidetes (**c**), and F/B ratio (Firmicutes and Bacteroidetes ratio) (**d**) after 10 weeks. The values were expressed as the mean ± S.E.M. * *p* < 0.05 and ** *p* < 0.01.

As a result of the relative abundance of bacterial taxonomy at phylum level, only two of the major phyla bacterial communities were detected (i.e., Firmicutes and Bacteroidetes, Figure 5a). PHGG intake in both dietary conditions induced a decrease in the abundance of phylum Firmicutes (CDC vs. GCDC: *p* < 0.01, CDW vs. GCDW: *p* < 0.01, HFDC vs. GHFDC: *p* < 0.05, and HFDW vs. GHFDW: *p* < 0.01). In addition, wheel running in PHGG treated mice under both dietary conditions (GCDW and GHFDW) induced a decrease in the abundance of Firmicutes (*p* < 0.01 and *p* < 0.05, Figure 5b). The phylum Bacteroidetes in HFDC mice was significantly higher than that in CDC mice (*p* < 0.05). Although in HFD conditions, PHGG and wheel running did not affect the abundance of Bacteroidetes, in CDC conditions, the Bacteroidetes was significantly increased by wheel running (*p* < 0.05), and its level of abundance was accelerated by wheel running under the PHGG intake (*p* < 0.01, Figure 5c). PHGG induced attenuation of the phylum Firmicutes and Bacteroidetes ratio (F/B ratio) in the control diet (CDC vs. GCDC: *p* < 0.01). Moreover, the F/B ratio was attenuated by PHGG intake while wheel running under both diet feedings. Interestingly, the F/B ratio was significantly decreased while wheel running in PHGG treated mice under both diet feedings (GCDC vs. GCDW: *p* < 0.01, and GHFDC vs. GHFDW: *p* < 0.01, Figure 5d).

**Figure 6:**
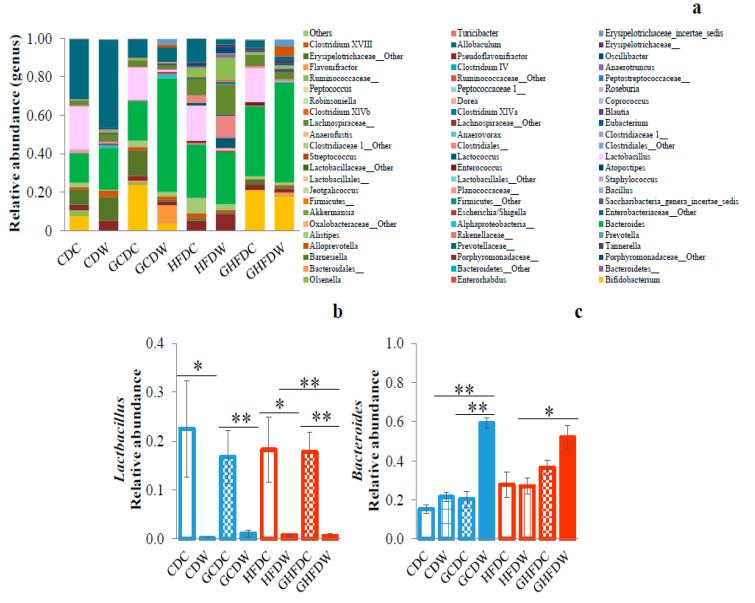
The supplemental effect of wheel running on gut microbiota at genus level in PHGG intake mice under CD and HFD-feeding. Relative abundance at genus level (**a**), Lactobacillus (**b**), and Bacteroides (**c**) after 10 weeks. The values were expressed as the mean ± S.E.M. * *p* < 0.05 and ** *p* < 0.01.

In Figure 6, the taxonomic composition of gut microbiota at genus level was shown (Figure 6a). Especially, we observed the decreased Lactobacillus abundance of wheel running in PHGG and non-PHGG intake mice under both diet feedings, but not PHGG intake only (Figure 6b). On the other hand, the genus Bacteroides abundance in GCDW mice was significantly higher than that in both CDW and GCDC groups (*p* < 0.01, respectively). Moreover, Bacteroides abundance in GHFDW mice was significantly higher than that in HFDW mice (*p* < 0.05, Figure 6c).

**Figure 7:**
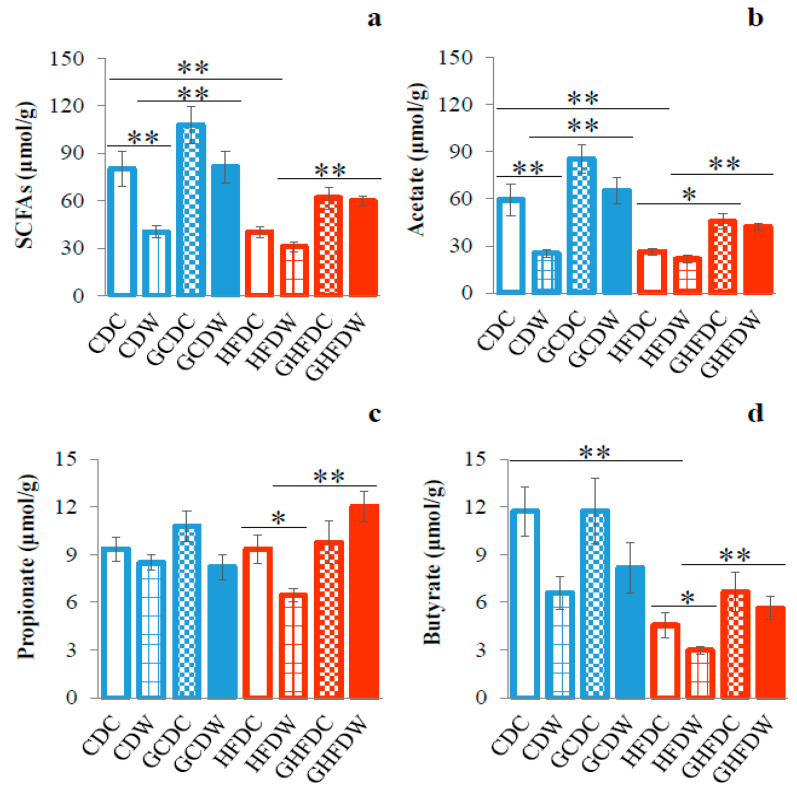
The supplemental effect of wheel running on cecum SCFAs in PHGG intake mice under CD and HFD feeding. The total SCFAs (**a**), acetate (**b**), propionate (**c**), and butyrate (**d**) contents in cecum were shown. The values were expressed as the mean ± S.E.M. * *p* < 0.05 and ** *p* < 0.01.

The amount of SCFAs (acetate, propionate and butyrate) in cecum contents was determined by HLPC analysis (Figure 7). The total SCFAs in HFDC mice was significantly lower than that in CDC mice (*p* < 0.01, Figure 7a). Especially, acetate (*p* < 0.01) and butyrate (*p* < 0.01) were attenuated by HFD (Figure 7b,d). In the control diet, exercise inhibited SCFAs (*p* < 0.01), especially acetate (*p* < 0.01), in cecum contents (Figure 7a,b). Moreover, in HFD, exercise inhibited propionate (*p* < 0.05) and butyrate (*p* < 0.05) in cecum contents (Figure 7c,d). In exercise groups, however, PHGG treatment accelerated the SCFAs increase in cecum contents ((SCFAs and acetate: CDW vs. GCDW and HFDW vs. GHFDW), and (propionate and butyrate: HFDW vs. GHFDW)).

## 4. Discussion

In this study, we expected that PHGG intake might be supplemented by voluntary exercise because PHGG, which is noviscous in form, is losing its functionary glycemic control [3]. Indeed, voluntary exercise in PHGG treated mice induced the attenuation of body mass gain and fat accumulation. Moreover, the results of GTT showed that BGAUC in GHFDW was lower than that in GHFDC mice. These results suggest that voluntary exercise might have a supplemental effect on obesity and insulin resistance in PHGG intake in mice. In fact, den Besten et al. [29] reported that supplements of guar gum markedly increased peripheral glucose clearance in HFD mice. Dietary PHGG, however, did not significantly affect plasma glucose levels in a rodent model of type 2 diabetes and patients with type 2 diabetes and the metabolic syndrome [4,5]. Therefore, exercise may be able to attenuate the abolished beneficial effect of glycemic control when the guar gum is hydrolyzed to a nonviscous form [3].

Here, we confirmed that cecum content weights were higher and the fecal pH was lower in mice fed with a HFD diet with PHGG than in those fed diets without PHGG. There was agreement with the results of a previous study, which suggested that the possible mediators of the effects of PHGG were the SCFAs produced by microbial fermentation of PHGG in the large intestine [13]. To date, the many findings of animal and human studies suggested that exercise may modulate the community and function of gut microbiota [16,17]. In the present study, there were almost no changes of α-diversities in CD mice. In contrast, HFD fed mice showed that almost all indexes were greatly affected by PHGG and wheel running exercise (Figure 4). Therefore, it was suggested that α-diversity, which means bacterial richness, evenness, and diversity, is firstly destabilized by HFD, and then is secondly affected by dietary fiber and/or exercise. In fact, in a HFD condition, a significant increase in α-diversities of gut microbiota was observed with voluntary exercise, but it was inversely decreased by exercise in PHGG-treated mice. Moreover, the β-diversity of gut microbiota showed that between the conditions of with and without PHGG treatment, but not exercise, was separated by cluster analysis. These results suggest that at least a presence or absence of PHGG intake makes for a completely different effect of exercise on the diversity of gut microbiota.

Obesity affects the diversity of the gut microbiota [30], and HFD feeding increases the abundance of Firmicutes and decreases the abundance of Bacteroidetes, which also means an increase in F/B ratio in gut microbiota [20,21,22,31,32]. Weitkunat et al. [12] reported that the abundances of Firmicutes and Bacteroidetes in HFD feeding mice did not change with or without guar gum intake. Our results also showed that the relative abundances of Bacteroidetes and F/B ratio showed no differences between HFDC and GHFDC mice, although Firmicutes was decreased by PHGG. However, voluntary exercise accelerated the decrease in the F/B ratio in PHGG intake mice. In fact, a human study showed the relationship between $\overset{\cdot }{V}$O_2_max and F/B ratio [33], and an animal experiment showed that Juvenile onset exercise increased Bacteroidetes and decreased Firmicutes [34]. In contrast, several studies also have reported that wheel running did not affect [34,35,36] or increase the F/B ratio [37,38]. Therefore, the suppression of weight gain by only recommended exercise habits might not necessarily lead to a change through the improvement of gut microbiota, and simply as F/B ratio. Therefore, our results suggest that combined with exercise, dietary fiber greatly induces decreases in the ratio of the F/B, thus improving gut dysbiosis.

Moreover, we observed decreases in the Lactobacillus abundance by voluntary exercise. It was reported that obesity was associated with a high level of the genus Lactobacillus [39]. It already showed that specific enzymatic activities of obese individuals were found in the Lactobacillus spp. in Firmicutes phylum rather than in Bacteroidetes [40]. Indeed, in a systematic review in humans and animals, it was found that the manipulation of the gut microbiota by *Lactobacillus acidophilus*, *L. ingluviei* or *L. fermentum* results in weight gain whereas specific strains of *L. gasseri* and *L. plantarum* used as food supplements presented an anti-obesity effect [41]. These findings suggest that voluntary exercise might have a protective effect on obesity via changes of gut microbiota in PHGG intake mice.

In contrast, PHGG intake mice increased the abundance of acetate-producing bacteria such as the genus Bacteroides [42,43], under voluntary exercise habits, and was consistent with the changes of total SCFAs and acetate in this study. Acetate constitutes the major part of the SCFAs which are fermented by gut microbiota [26,44], might induce not only suppression of body fat accumulation in obese or diabetic animals, but also promote anti-inflammation [45,46,47]. Moreover, it was already known that PHGG could increase fecal moisture and small intestinal transit and shortened the time to first black stool defecation after constipation. It also predominantly promotes the accumulation of Bacteroidetes [6].

A problem at consumption of raw guar gum is that it rapidly forms a tight gel, rendering it unpalatable when hydrated. In order to make guar gum more palatable, manufacturers have produced PHGG, a low viscosity/nonviscous [3]. However, PHGG renders the guar gum ineffective for viscosity dependent health benefits like an improved glycemic control. Therefore, it may be useful to consider exercise habits combined with intake of PHGG.

In summary, our data showed that voluntary exercise in PHGG intake mice induced not only the attenuation of body mass gain and fat accumulation, but also improvement of glucose metabolism. In addition, the supplemental effect of voluntary exercise on gut microbiota, which decreased the ratio of the F/B ratio, in PHGG intake under high-fat diet feeding was observed. Taken together, combined with exercise, PHGG intake might improve obesity and gut microbiota composition in HFD-fed mice.

## Supplementary Materials

The following are available online at https://www.mdpi.com/2072-6643/12/9/2508/s1, Figure S1: Schematic illustration of experimental design of this study, Figure S2: Effects of PHGG and wheel running on body mass (a) and food intake (b) in CD and HFD fed mice.
