# Supplementary material for: The Effect of Voluntary Exercise on Gut Microbiota in Partially Hydrolyzed Guar Gum Intake Mice under High-Fat Diet Feeding
Source: Nutrients. 2020 Aug 19;12(9):2508. doi: 10.3390/nu12092508 (PMC7551544; doi:10.3390/nu12092508)
Supplement: Supplementary file 1 [file nutrients-12-02508-s001.pdf]

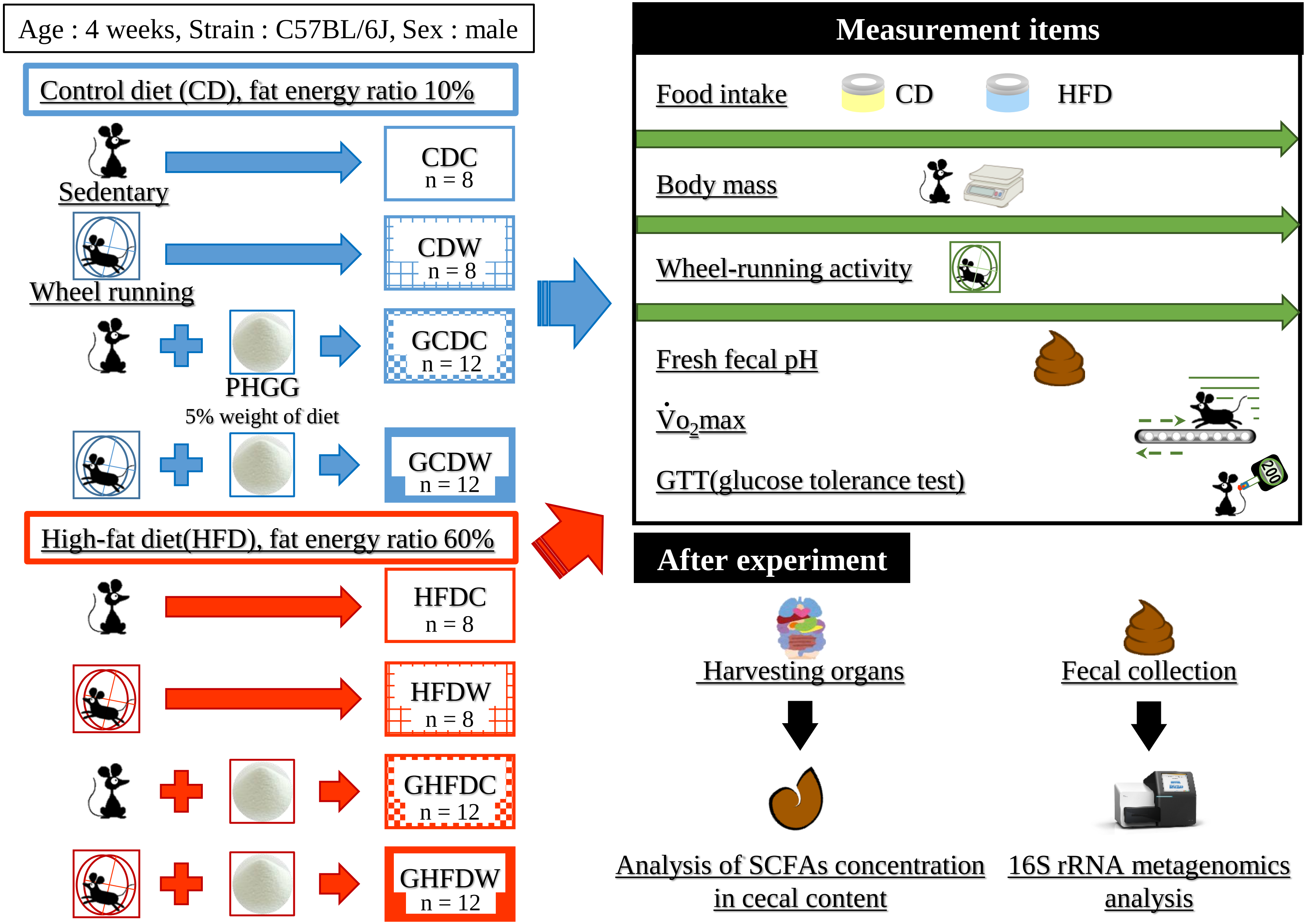

Figure S1. Schematic illustration of experimental design of this study.

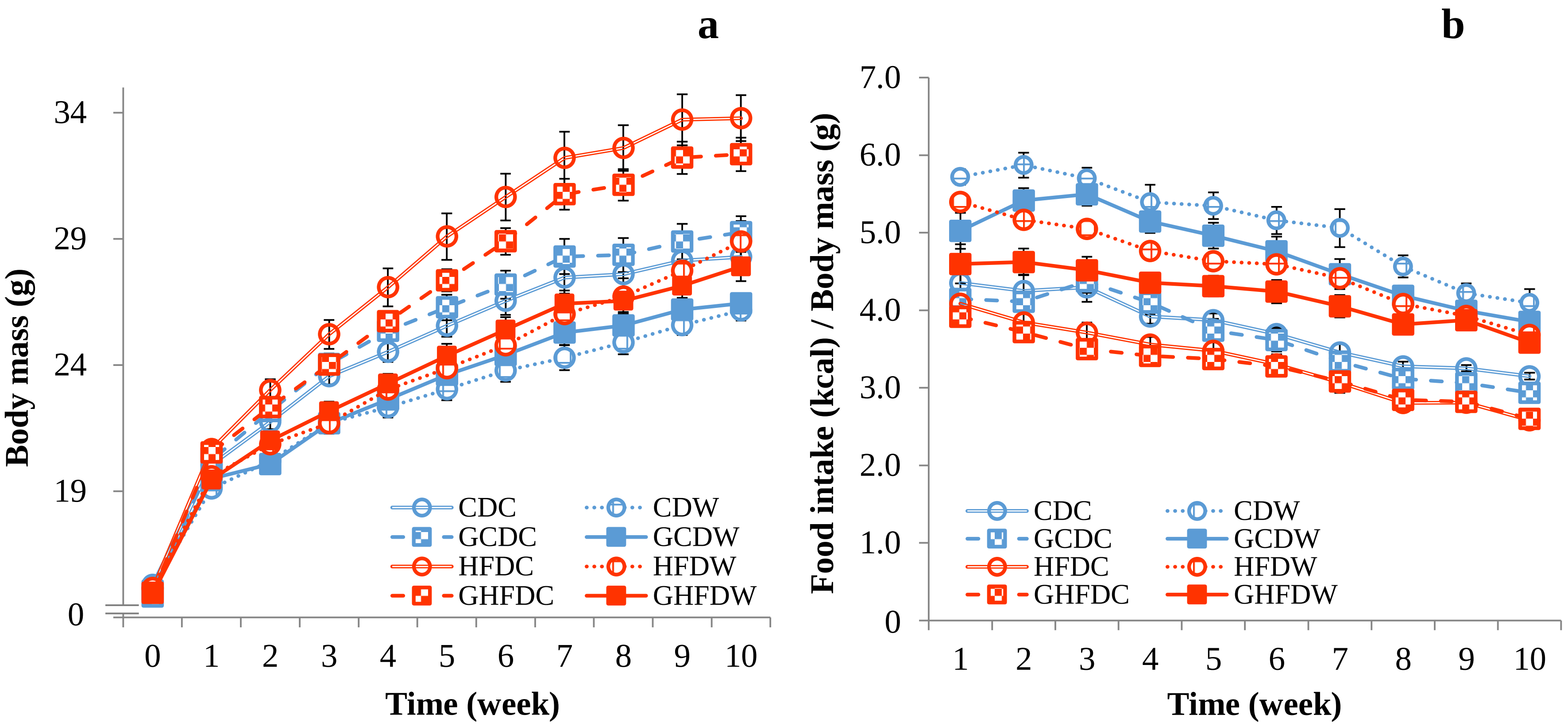

Figure S2. Effects of PHGG and wheel running on body mass (a) and food intake (b) in CD and HFD fed mice.

Food intake showed as energy intake (kcal) per body mass at each week. The values were expressed as the mean ± S.E.M.
